# Drug Utilization Research and Predictors of Outcomes in the Intensive Care Unit of a Tertiary Care Hospital: A Prospective Observational Study

**DOI:** 10.7759/cureus.50653

**Published:** 2023-12-17

**Authors:** Niti Mittal, Monika Verma, Shaveta Siwach, Priyanka Bansal, Suresh Kumar Singhal

**Affiliations:** 1 Pharmacology and Therapeutics, Pandit Bhagwat Dayal Sharma Post Graduate Institute of Medical Sciences, Rohtak, IND; 2 Pharmacology, Pandit Bhagwat Dayal Sharma Post Graduate Institute of Medical Sciences, Rohtak, IND; 3 Critical Care, Pandit Bhagwat Dayal Sharma Post Graduate Institute of Medical Sciences, Rohtak, IND; 4 Anaesthesiology, Pandit Bhagwat Dayal Sharma Post Graduate Institute of Medical Sciences, Rohtak, IND

**Keywords:** antibiotic prescriptions, who indicators, polypharmacy, mortality, predictors, prescribed daily dose, defined daily dose, intensive care

## Abstract

Background: Multiple drugs are commonly prescribed to intensive care unit (ICU) patients owing to the disease profile, multiple organ dysfunction, prophylaxis, management of stress ulcers, nosocomial infections, etc. This study aimed to evaluate the drug utilization patterns and factors influencing mortality and duration of stay in ICU patients.

Methodology: A prospective observational study was conducted in the ICU of our tertiary care hospital, Postgraduate Institute of Medical Sciences, Rohtak. Data was collected from treatment charts of patients using a structured pretested proforma. World Health Organization Anatomical Therapeutic Chemical/Defined Daily Dose (WHO ATC/DDD) methodology and core prescribing indicators were used to assess drug utilization data. The effect of different variables on mortality and duration of stay in the ICU was evaluated using regression analysis.

Results: An average of 8.78 drugs were prescribed per patient. Among the 922 prescriptions, anti-infectives, anti-inflammatory drugs, and drugs acting on the gastrointestinal tract were the most frequent medication classes prescribed. Polypharmacy and trade name prescribing were common. For most of the drugs, the prescribed daily dose corresponded to the WHO-DDD except ceftriaxone and levofloxacin. Age, presence of cardiac disorders, and Glasgow Coma Scale (GCS) score at admission directly correlated with mortality while the use of diuretics had a negative correlation with the duration of ICU stay.

Conclusions: There is a need to rationalize drug therapy in the ICU with regard to limiting polypharmacy and emphasizing generic drug name prescribing and adherence to the essential drug list. Antibiotic prescription patterns, in particular, deserve a special focus keeping in mind the multitude of factors demanding aggressive antibiotic use in critically ill intensive care patients.

## Introduction

Drug utilization research is defined as “the marketing, distribution, prescription, and use of drugs in a society, with special emphasis on the resulting medical, social, and economic consequences” [1]. Drug utilization research studies conducted in various hospital settings serve as valuable tools in assessing drug prescribing patterns; analyzing hospital drug policies; and making recommendations to improve drug usage trends in the future, if needed.

Intensive care units (ICUs) care for critically ill patients and a number of drugs are used for the treatment of such patients. Despite the existence of various guidelines regarding drug usage in ICUs, sometimes their implementation might be challenging owing to factors such as patient-specific illnesses, resident learning curves, and physician drug preferences [2]. The incidence of medication errors in ICUs is also high owing to the huge workload. In a prescription analysis study in adult ICUs, 73.26% drug formulation and 40% dosage strength-related omissions were revealed in the prescriptions [3]. In a multicentric study in adult ICUs in the USA, it was reported that around half of the antibiotic prescriptions were administered for a prolonged duration than indicated thereby predisposing to increased risk of resistance [4]. Ali et al. in a study conducted on 100 ICU patients observed 86% irrational antibiotic prescriptions and among them 96.5% mortality [5]. Another four-year prospective longitudinal study including 8,763 patients reported that 42% of antimicrobial prescriptions were inappropriate [6]. Few other drug utilization studies conducted in ICU settings reported antimicrobials as the most commonly prescribed agents [7,8].

Some previous studies have explored the association between various covariates and clinical outcomes as well as the duration of stay in ICUs. The total number of prescriptions and use of antibiotics, insulin, or inotropes were reported as significant predictors of mortality [9-11]. Bobek et al. conducted an exploratory multiple regression analysis using the length of stay as the dependent variable and the following as independent variables: Acute Physiology and Chronic Health Evaluation (APACHE III) scores, gender, age, death, occurrence of hospital-acquired infections, self-extubation, use of physical restraints, and number of medication classes received. It was revealed that the number of medication classes was a significant independent factor associated with the length of stay [2].

With this background, the present study aimed to evaluate drug utilization patterns among patients admitted to ICUs and to identify the factors influencing mortality and duration of stay in ICUs. The study findings are expected to identify potential areas demanding improvement in drug prescribing patterns in ICUs and further hospital drug dispensing policies.

## Materials and methods

Study design and setting

This was a prospective observational longitudinal cohort study conducted collaboratively by the Departments of Pharmacology and Anaesthesiology, Pandit Bhagwat Dayal Sharma Postgraduate Institute of Medical Sciences, Rohtak. Ours is a public tertiary care institute in the North Indian state of Haryana, India with 0.4-0.5 million annual admissions. The institute has well-equipped ICUs under various medical, surgical, and critical care departments.

Study inclusion and exclusion criteria

For the present study, modular ICUs (20 ICU beds) under the Department of Anaesthesia and Critical Care were included. The inclusion criteria were (i) patients admitted in included ICUs during the study period, (ii) any age, (iii) either male or female, and (iv) who gave written informed consent to participate in the study. The exclusion criteria were patients who refused to give consent. 

Study period

The study was conducted over a period of six months from September 2022 to February 2023. During this period, all the included patients were followed up for their entire duration of stay in ICUs. 

Data collection and management

Data was collected by two postgraduate residents trained *a priori *regarding* *data extraction and collection.* S*tructured pretested proforma designed by the Principal Investigator was used to gather information pertaining to patient demographics, reason for ICU admission, disease severity (assessed by Glasgow coma scale and Acute Physiology And Chronic Health Evaluation: APACHE III score), co-morbid conditions, indwelling devices, medication details, duration of stay in ICUs, and outcome. Data was extracted from case files and treatment charts and any desired missing information was obtained from the treating physicians.

Various drug utilization metrics were analyzed in accordance with the World Health Organization Anatomical Therapeutic and Chemical Classification/ Defined Daily Dose (WHO ATC/DDD) methodology [12]. All the drugs were coded as per the WHO-ATC coding system. Different parameters such as the percentage of drugs prescribed, defined daily dose (DDD), DDD/100 bed days, and prescribed daily dose (PDD) were analyzed. From the prescription data in the included treatment charts, the amounts of drugs consumed were converted into the number of defined daily doses (DDD) as per the formula:

Number of DDDs = Number of items issued x amount of drug per item / WHO DDD measure

The data was represented as DDDs per 100 bed days. The estimated PDD was calculated by multiplying DDD with the ratio of the number of DDDs to the number of treatment days. The prescriptions were also analyzed for WHO core prescribing indicators for drug utilization research.

Statistical analysis

Data was expressed as Mean ± SD, median (inter-quartile range), and numbers (percentages). All the data were entered into a master chart using a Microsoft Excel sheet and subjected to statistical analysis. The correlation between the number of prescriptions and various patient demographic characteristics (age, gender, presence of comorbidity, admission diagnosis, GCS score at admission, APACHE III score, and duration of ICU stay) was assessed using linear regression analysis. Binary logistic regression and linear regression analysis were used to predict the effect of different variables (age, gender, admission diagnosis, disease severity at admission, presence of comorbid illness/es, total number of drug prescriptions, and various classes of drugs prescribed) on mortality and duration of stay in ICU, respectively. All the analyses were carried out using IBM SPSS Statistics for Windows, Version 28 (Released 2021; IBM Corp., Armonk, New York, United States). A P-value < 0.05 was considered statistically significant.

Ethical considerations

The study was approved by the Biomedical Research Ethics Committee of PGIMS, Rohtak (BREC/21/115). Adequate measures were taken to ensure confidentiality and only de-identified data were used.

## Results

During the study period, 105 patients were admitted in the included ICUs all of whom consented to participate. Hence, the study population comprised 105 participants. 

Study population demographics

Characteristics of the study population are depicted in Table 1. The study population comprised 65 (61.9 percent) males and had a mean age of 42.9 years. The patients were admitted for various diagnosis most common being trauma and surgical. The mean duration of stay in the ICU was 7.9 days. Eighty-two (78 percent) patients were on mechanical ventilation.

**Table 1:** Demographic and other characteristics of study subjects during ICU stay (N=105). *Snakebite, hanging, hyperkalemia, diabetic complications; SD: Standard deviation; CNS: central nervous system; GCS: Glasgow Coma Scale; APACHE: Acute Physiology And Chronic Health Evaluation

Characteristics	
Males, n (%)	65 (61.9)
Age in years, Mean (SD)	42.9 (19.6)
Admission diagnosis, n (%)	
Trauma	31 (29.5)
Surgical	29 (27.6)
Obstetric disorders	10 (9.5)
Respiratory disorders	9 (8.6)
Poisoning	9 (8.6)
CNS disorders	8 (7.6)
Cardiac disorders	3 (2.8)
Others*	7 (6.7)
Duration of ICU stay in days, Mean (SD)	7.8 (9.9)
Co-morbidities, n (%)	
Present	66 (62.8)
Absent	39 (37.1)
Severity of illness	
GCS score, Mean (SD)	9.8 (4.9)
APACHE III score, Mean (SD)	14.7 (9.9)
On mechanical ventilation, n (%)	82 (78)
Drug classes prescribed, n (%)	
Antibiotics	105 (100)
Anti-ulcers	102 (97)
Respiratory	79 (75)
Corticosteroids	57 (54.3)
Nutritional	54 (51)
Diuretics	40 (38)
Antiepileptics	44 (41.9)
Outcome, n (%)	
Shifted	60 (57.1%)
ICU mortality	45 (42.8%)

Prescription pattern of drugs

A total of 922 prescriptions were recorded during the study period. The number of medications prescribed per patient ranged from 4 to 18 (median: 5.82, mean ± SD: 8.78 ±2.86). All patients except one received five or more drugs. Major classes of drugs prescribed in ICU are shown in Table 2. 

**Table 2:** Proportional prescription of various classes of drugs in the ICU.

Class of drugs	Number of prescriptions (%)
Antiinfectives	232 (25.16)
Gastrointestinal tract drugs	129 (14)
Cardiovascular drugs	90 (9.76)
Antiinflammatory	170 (18.44)
Central Nervous System drugs	79 (8.56)
Drugs affecting blood	30 (3.25)
Respiratory drugs	49 (5.3)
Nutritional supplements	64 (6.94)
Others	79 (8.56)

The number and classes of drugs prescribed varied depending on patient profiles and indications. In linear regression analysis, there was a lack of correlation between various covariates (age, gender, presence of comorbidity, admission diagnosis, GCS score at admission, APACHE III score, and duration of ICU stay) and the total number of prescriptions.

The most frequent classes of medications prescribed were anti-infectives (232 (25.16%)] followed by anti-inflammatory drugs [170 (18.44%)] and drugs acting on the gastrointestinal tract (GIT) [129 (14%)). Beta-lactam antibiotics (123/232; 53%) and antiprotozoal agents (metronidazole) (57/232; 24.5%) were most commonly prescribed among anti-infectives. Anti-inflammatory drugs prescribed included non-steroidal anti-inflammatory drugs (NSAIDs) (98/170; 57.6%) and corticosteroids (72/170; 42%) while among the drugs acting on GIT, proton pump inhibitors (100/129; 77.5%) and antiemetics (ondansetron, metoclopramide) (24/129; 18.6%) were the frequently used agents. Fixed drug combinations (FDCs) accounted for 9.97 percent of the total prescriptions (92/922) and mainly comprised antibiotics (beta-lactam/ beta-lactamase inhibitor) and anti-asthmatic (levo-salbutamol/ ipratropium) drug combinations. 

All patients received antibiotic/s out of which 22 (21%) were single antibiotic prescriptions while 54 (51%) and 29 (28%) had two and three or more antibiotics prescribed, respectively.

WHO core prescribing indicators

All the patients (105; 100%) received injectables and antibiotics during the ICU stay. An average of 8.78 drugs per prescribed per patient. Out of a total of 922 prescriptions, 783 (84.9%) were from NLEM 2022 while 545 (59%) were by generic names. The majority of the nutritional (48/55; 87.3%), anti-inflammatory (77/89; 86.5%), and CNS drugs (44/55; 80%) were prescribed by generic names while trade name prescriptions were mainly observed for respiratory (41/43; 95%) and GIT drugs (108/115; 94%).

Drug utilization metrics

The total number of DDDs consumed was maximum for antiprotozoals, beta-lactam antibiotics, and proton pump inhibitors while the number of DDDs per 100 bed days was higher for antiprotozoals, corticosteroids, and calcium channel blockers (Table 3).

**Table 3:** Comparison of data on defined daily doses of major groups of drugs prescribed. GIT: gastro-intestinal tract; PPIs: proton pump inhibitors; NSAIDS: non-steroidal anti-inflammatory drugs; CCBs: calcium channel blockers; *Adrenergics in combination with corticosteroids or other drugs, excluding anticholinergics (WHO ATC/DDD index 2023); #Angiotensin converting enzyme (ACE) inhibitors, alpha blockers, alpha agonists, anti-psychotics, laxatives; ATC: Anatomical Therapeutic Chemical; DDD: defined daily dose

Major group	Class	ATC Code	No. of DDDs	No. of bed days	DDDs per 100 bed days
Antibacterials	Beta lactams	J01C	661.45	435	152
	Imidazole derivatives	J01XD	2181.67	220	991.67
	Aminoglycosides	J01G	135	96	141
	Macrolides	J01F	14	15	46
	Tetracyclines	J01A	74	37	20
Drugs acting on GIT	PPIs	A02BC	587	501	117.16
	Antiemetics	A04	99.62	67	148.68
Anti-inflammatory	NSAIDS	M01A	158.6	275	57.67
	Corticosteroids	H02	413.6	106	390.1
Cardiovascular drugs	Diuretics	C03	107.4	149	72.08
	CCBs	C08	146	61	239.34
	Statins	C10AA	70.75	35	202
	Aspirin	B01AC06	24	24	100
Central Nervous System drugs	Anti-epileptics	N03A	68	127	53.54
	Opioids	N02A	113.4	92	123.2
Respiratory system drugs	Bronchodilators*	R03AK	37	247	14.9
Others^# ^			1661	60	2768

The PDD of all the drugs prescribed was in accordance with WHO DDD except for ceftriaxone and levofloxacin for which the PDD was on a higher side (Table 4).

**Table 4:** Comparison of estimated prescribed daily doses and defined daily doses of drugs from highly prescribed groups. ATC: Anatomical Therapeutic Chemical; DDD: defined daily dose; PDD: prescribed daily dose

Class	Individual drug	ATC Code	No. of DDDs	No. of bed days	DDDs per 100 bed days	WHO DDD	PDD
Beta lactams	Ceftriaxone	J01DD04	492.75	226	218	2g	4.36
	Meropenem	J01DH02	65.5	104	62.9	3g	1.88
	Piperacillin-tazobactam	J01CR05	76	114	66.6	14g	9.3
	Amoxicillin-clavulanate	J01CR02	27.2	31	87.74	3g	2.63
Glycopeptides	Vancomycin	J01XA01	7.5	24	31.25	2g	0.62
Aminoglycosides	Amikacin	J01GB06	135	96	141	1g	1.41
Macrolides	Azithromycin	J01FA10	14	15	93.3	0.5g	0.46
Quinolones	Levofloxacin	J01MA12	41.5	15	276.6	0.5g	1.3
Imidazoles	Metronidazole	J01XD01	2181.6	220	991.67	1.5 g	1.485
Proton pump inhibitors	Pantoprazole	A02BC02	587	501	117.16	40mg	46.8

Predictors of mortality and duration of stay in the ICU

A total of 45 patients out of 105 died during ICU stay. Out of the various determinants, age, presence of cardiac disorders, and GCS score at admission directly correlated with mortality while there was a lack of correlation of mortality with the total number of prescriptions and various classes of drugs prescribed. Among the covariates assessed for the duration of ICU stay, only the use of diuretics (Odds ratio (95% CI): -4.19 (-8.37, -0.005); P value: 0.05) was found to have a significant negative correlation with the duration of ICU stay (Table 5).

**Table 5:** Binary logistic regression analysis of predictors of mortality among ICU patients. GCS: Glasgow Coma Scale; APACHE: Acute Physiology And Chronic Health Evaluation: NSAIDs: non-steroidal anti-inflammatory drugs. P value<0.05 was considered statistically significant.

Variable	Odds ratio (95% C.I.)	P value
Age	0.95 (0.92, 0.99)	0.01
Gender		
Male	Reference	
Female	1.44 (0.39, 5.28)	0.58
Admission diagnosis		
Trauma	Reference	
Surgical	1.79 (0.45, 7.09)	0.4
Respiratory disorders	1.88 (0.19, 17.93)	0.58
Poisoning	2.41 (0.32, 18)	0.39
CNS disorders	1.69 (0.18, 15.79)	0.64
Obstetric disorders	0.14 (0.01, 1.59)	0.11
Cardiac disorders	41.68 (1.21, 1432.3)	0.03
Others	3.4 (0.2, 56.18)	0.39
Comorbidity (Yes/No)	0.83 (0.25, 2.73)	0.77
GCS score	1.26 (1.08, 1.48)	0.003
APACHE score	0.93 (0.84, 1.04)	0.25
Total prescriptions	0.93 (0.77, 1.12)	0.47
NSAIDs (Yes/No)	0.85 (0.18, 3.83)	0.83
Steroids (Yes/No)	0.88 (0.29, 2.65)	0.82
Antiepileptics (Yes/No)	0.78 (0.25, 2.42)	0.67
Diuretics (Yes/No)	0.52 (0.17, 1.53)	0.23
Bronchodilators (Yes/No)	1.34 (0.45, 4)	0.59
Antiplatelets (Yes/No)	1.97 (0.51, 7.53)	0.32
Constant	3.48	0.52

## Discussion

The present drug utilization study was carried out in the ICU under the Department of Anaesthesia and Critical Care in our tertiary care hospital. During the six-month study period, a total of 105 admitted patients were followed up and included in the analysis. Congruent to similar studies in the literature [7,11,13,14], the study population comprised more males and had a mean age of 42.9 years. 

In our study, an average of 8 with a maximum of 18 drugs were prescribed per patient which was comparable or lower to earlier studies conducted in similar settings from India as well as abroad [7,11,15,16]. Polypharmacy (five or more drugs prescribed) was observed (99%) which was in line with some earlier studies from India [17-20]. Panamanian et al., however, reported only 57% incidence of polypharmacy [21]. Management of critically ill patients demands aggressive prophylactic and therapeutic measures. Certain drugs routinely used prophylactically in ICU settings are anti-ulcers, sedatives, deep vein thrombosis (DVT) prophylaxis, antibiotics for the prevention of nosocomial infections, and anti-reflux agents. Under certain circumstances and depending on patient profile characteristics, polypharmacy may be deemed imperative for example empirical prescriptions in case of indefinite diagnosis at admission, presence of multiple comorbidities, disease severity, multiple organ dysfunction, etc. In our study, we did not observe any association of the total number of prescriptions with various covariates such as age, gender, presence of comorbidity, admission diagnosis, GCS score at admission, APACHE III score, and duration of ICU stay. Few earlier studies though reported a correlation of a total number of drugs with disease severity and diagnosis at admission [9,22]. Nonetheless, it is always ideal to maintain the average number of pills per prescription as low as possible in order to save treatment costs and lower the risk of unwanted adverse effects and drug-drug interactions. Indeed, more than 10 percent of preventable adverse events among ICU patients were attributed to drug-drug interactions, a repercussion of polypharmacy [23-25]. 

The most commonly prescribed group of drugs included anti-infectives and drugs acting on the GIT, a finding concordant with many earlier studies [9,11,17-20]. All the patients had encounters with one or more antibiotics during their ICU stay. In view of critical illnesses, a long period of stay in ICU, multiple organ dysfunction, and unconscious status, patients admitted to ICU are 5 to 10 times more vulnerable to nosocomial or healthcare-associated infections (HAIs) compared to those in general wards [26] which were reported to affect about 30 percent of ICU patients [5], a fact partly justifying the higher use of antibiotics in ICU settings. Seventy-nine percent of our study population received more than one antibiotic agent, a finding in agreement with similar studies from India and other developing countries [7-9,11,17-22]. This is, however, in contrast to data from ICUs in Western countries reporting a relatively lesser proportion of patients being prescribed multiple antibiotic agents [11,27,28]. Prolonged mechanical ventilation and long duration of ICU stay are associated with ventilator-associated pneumonia (VAP). 78 percent of our patients were intubated which also probably explains the high antibiotic consumption in the present study.

More than 25 percent of our patients had undergone surgery. Depending upon the type of surgery (clean/ contaminated/ dirty), choice and duration of antibiotic prophylaxis are routinely recommended to prevent surgical site infections (SSIs). Post-operative fever is another indication for the continuation of an antibiotic regimen after completion of surgery [29]. Besides these, the empirical prescription of antibiotics especially in a tertiary care referral hospital like ours may be partly explained by the high rate of emergency surgeries and cases where the probability of infection cannot be ruled out. Notwithstanding such indications, the fact that extensive and inappropriate use of antibiotics is associated with the emergence of multidrug-resistant organisms cannot be ignored. Hence, it is crucial to optimize the administration of antibiotics in ICU settings. Beta-lactams comprised more than half of the total antibiotic prescriptions, the most common agents being third-generation cephalosporins (ceftriaxone) and beta-lactam/beta-lactamase inhibitors. Due to the wide spectrum of action, favorable safety profile across different age groups, and cost, this group of antibiotics is particularly preferable in ICU patients who are prone to multiple infections due to indwelling devices. In fact, third-generation cephalosporins were the most frequently prescribed antibiotics in various other similar studies [7,9,11,13-21]. 

An overuse of pantoprazole (97% of patients) was observed, a finding in agreement with earlier studies [11,13-21]. Proton pump inhibitors reduce stomach acid output, inhibit upper gastrointestinal hemorrhage, and enhance mucosal healing. In fact, in a study conducted by Mohebbi and Hesch, 75 to 100 percent of ICU patients were reported with stress-related mucosal illness after 24 hours of admission suggesting the need for acid suppression medication for stress ulcer and gastrointestinal bleeding prevention in ICU settings [27].

The WHO core prescribing indicators measure the appropriateness of drug prescription practices by utilizing several key dimensions. Assessment of these indicators in our study has generated useful information reflective of the quality of health care delivered by the ICU in our hospital. 100 percent encounters with injectables and antibiotics were reported. The clinical profile of ICU patients usually necessitates the administration of drugs through the parenteral route due to the quick onset of action and ability to use in unconscious patients. About eighty-five percent of the drugs were prescribed from WHO Essential Medicine List 2021 which was higher than that reported earlier [9,11]. Nearly 60 percent of drugs were prescribed as generic names which was lesser than that reported earlier [3]. 

Previous studies have reported an association between various demographic variables and drug classes prescribed with the outcome as well as the duration of stay in the ICU. Our ICU patients had a mortality rate of 42.8 percent comparable to earlier studies in India [9,17]. Few studies from other developing nations reported mortality rates from 25 to 83 percent [5,11]. We did not observe any correlation of mortality with the number and classes of drugs prescribed. Biswal et al. reported that the requirement of insulin or inotropes signified an adverse outcome on mortality [9]. Hartmann et al. reported that antibiotic therapy can be viewed as a surrogate marker for infections and correlates with hospital mortality in patients staying for more than 24 hours in a surgical ICU [10]. Zakwani et al. also observed statistically significant associations between clinical outcome and the total number of prescriptions and duration of ICU stay though lack of correlation between clinical outcome with age and gender was reported, a finding in congruence with our study [11]. Another study reported significantly higher mortality rates in patients on ventilators and being prescribed steroids, irrational antibiotics, or nephrotoxic drugs [5]. In our study, it was observed that diuretic use was associated with shorter length of stay in ICU though there was a lack of correlation between various other covariates studied and duration of ICU stay. Bobek et al., in a similar analysis, reported an association between the number of drugs prescribed and the duration of stay in the ICU [2].

We acknowledge some limitations of our study. The information on the nature of antibiotic prescriptions (empiric/ prophylactic or definitive) could not be extracted, which was however beyond the scope of the present study which aimed to analyze drug prescription practices in ICU in general. However, our findings with respect to antibiotic prescription patterns demand diligent observation of various factors such as likely pathogens in specific settings, colonization with resistant organisms, patient-specific characteristics like antibiotic allergies, etc. An in-depth analysis of such aspects may probably elucidate the reasonable rationality or appropriateness of the number, regimens, and choice of antibiotic agents being prescribed. The study was also challenging in terms of the current non-existence of antibiotic policy and local antibiotic guidelines in our hospital which may strongly influence antibiotic use patterns, thus necessitating caution during interpretation of observations. Another drawback worth mentioning is that the study results have limited generalizability because data collection was confined to a single center with a sample representative of a very small proportion of the entire population. We also did not carry out an elaborate and discrete analysis for illnesses managed differently such as trauma, postoperative complications, cardiac disorders, poisoning, etc. which would probably give a clearer picture of the appropriateness of drug prescription patterns. Notwithstanding these limitations, the data generated from the present study can serve as a useful guide for comparison in future single or multicentric studies in this field.

## Conclusions

The application of elementary strategies of drug utilization has proved to be a convenient, effective, and inexpensive tool for wide-scale improvements in intensive care therapeutic practices. The present drug utilization study highlights the need for rationalizing drug therapy in intensive care settings with regard to limiting polypharmacy, emphasizing generic drug name prescribing, and increasing adherence to essential drug lists. Antibiotic use practices in ICUs remain far from the guidelines probably due to patients’ critical illnesses and physician preferences. Such circumstances call attention to various areas for improvement such as the establishment of antibiotic surveillance mechanisms; generation of high-quality evidence in the prevalent antibiotic use practices; formulation and implementation of hospital antibiotic guidelines and the development of multidisciplinary approaches for antibiotic management in critically ill intensive care patients.
